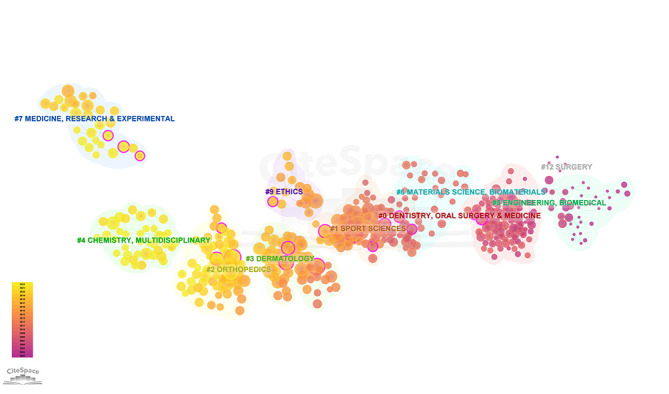# Platelet-rich plasma: A bibliometric and visual analysis from 2000 to 2022

**DOI:** 10.1097/MD.0000000000040530

**Published:** 2024-11-15

**Authors:** Kai Du, Ao Li, Chen-Yu Zhang, Ren Guo, Shu-Ming Li

**Affiliations:** a Beijing University of Chinese Medicine, Beijing, People’s Republic of China; b Department of Pain Medicine, Beijing Hospital of Traditional Chinese Medicine, Capital Medical University, Beijing, People’s Republic of China.

**Keywords:** bibliometric analysis, CiteSpace, platelet-rich plasma, visual analysis, VOSviewer

## Abstract

**Background:**

Platelet-rich plasma (PRP) is an integral biotherapeutic modality with evolving significance in the medical domain. Despite its expanding applications, a comprehensive bibliometric evaluation is essential to understand its development and impact.

**Methods:**

The Web of Science core collection subject search identified articles pertinent to PRP applications. Analytical tools, including CiteSpace, VOSviewer, Bibliometrix (R-Tool for R-Studio), TBtools, SCImago Graphica, Origin, and Excel, facilitated the bibliometric scrutiny. This examination spanned dimensions ranging from geographical and institutional contributions to thematic shifts and keyword prevalence.

**Results:**

A corpus of 5167 publications was analyzed, with the United States, particularly the Hospital for Special Surgery, emerging as major contributors. The American Journal of Sports Medicine was identified as the primary journal, and Anitua Eduardo as the leading author in the domain. Keyword analysis highlighted evolving research themes, with a shift from traditional applications in orthopedics and dentistry to emerging areas such as dermatology, aesthetics, and chronic pain management.

**Conclusion:**

The bibliometric analysis of PRP research reveals a multifaceted array of applications across various medical disciplines and highlights areas requiring further exploration, particularly in standardization, personalization, and safety. Future advancements in PRP research will necessitate innovative exploration, ethical considerations, and rigorous scientific validation to fully harness the therapeutic potential of PRP and related therapies.

## 1. Introduction

Platelet-rich plasma (PRP) is an autologous blood product with platelet concentrations significantly higher than those in normal circulating blood.^[1]^ These platelets contain α-granules packed with essential growth factors that promote tissue repair and regeneration. Since its emergence in the 1970s, PRP has become a cornerstone of regenerative medicine. Its applications range from promoting bone fracture healing and alleviating osteoarthritis symptoms to aesthetic procedures like skin rejuvenation. As medical science evolves, PRP continues to demonstrate its versatility and therapeutic significance across various specialties.^[2]^

Despite its growing popularity, PRP is controversial, particularly regarding its optimal platelet concentration. Although higher concentrations are often thought to enhance therapeutic efficacy, studies show a nonlinear relationship, with concentrations above a specific threshold not necessarily improving healing or regenerative outcomes.^[3–7]^ There are also debates on the composition of PRP, specifically between leukocyte-rich PRP and leukocyte-poor PRP, and their respective therapeutic benefits and potential side effects like inflammation.^[8–13]^ The role of erythrocytes in PRP is also contested. The challenge lies in optimizing the interplay between platelets, leukocytes, and erythrocytes to maximize PRP’s therapeutic efficacy.^[14]^

Variations in PRP preparation methods result in differing final compositions, and the absence of standardized protocols exacerbates inconsistencies in therapeutic outcomes. While PRP’s autologous nature generally ensures a favorable safety profile, risks such as post-procedure pain, swelling, and infection persist, particularly without standardized protocols. As PRP applications expand, reaching a consensus on these debates is crucial for its standardized and effective clinical use.

Bibliometrics, which involves the statistical analysis of written publications, provides nuanced insights into specific research areas by quantifying publication trends and mapping knowledge structures, revealing a field’s evolution and current state. This study presents a rigorous bibliometric assessment of PRP research over 2 decades, aiming to decode its multifaceted trajectory, identify influential contributors, and highlight emerging research themes.

## 2. Materials and methods

### 2.1. Search strategy

**Figure 1.**
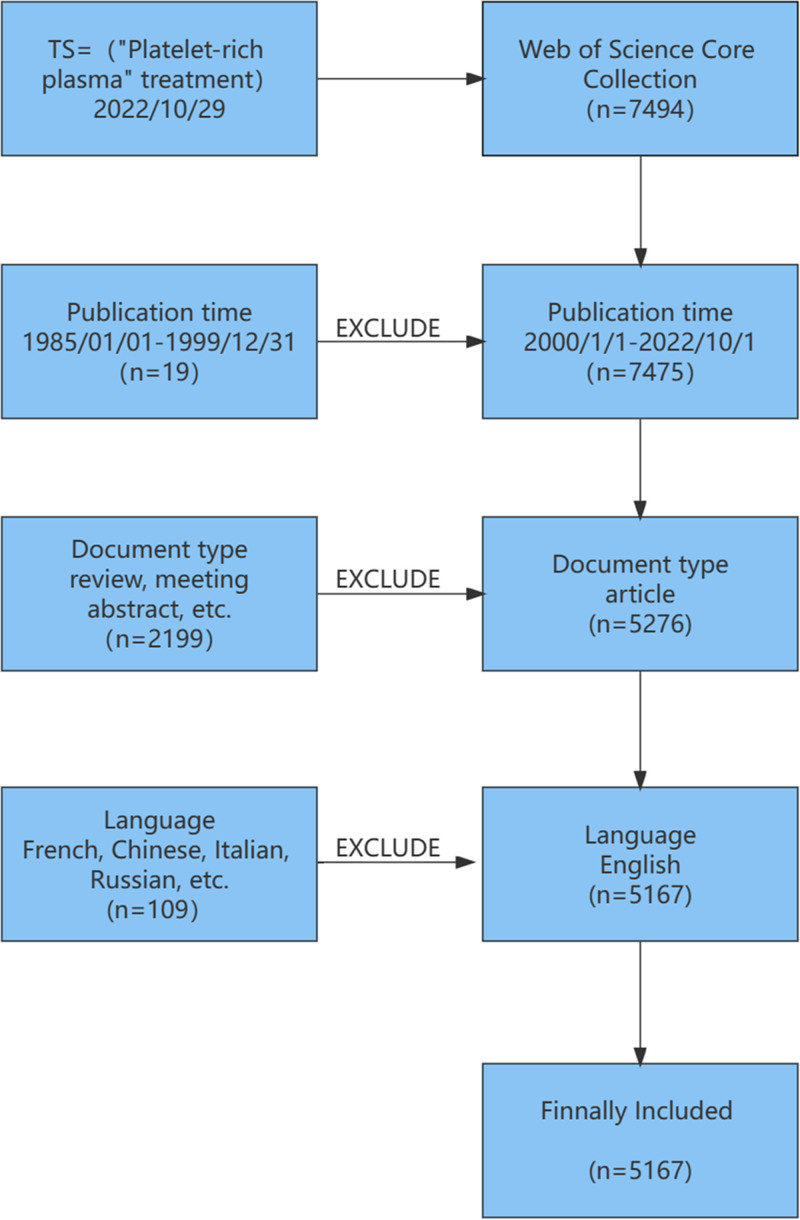
Literature screening process. A diagram illustrating the stages of literature selection, from initial search and filtering based on inclusion criteria to the final selection of articles relevant to PRP research.

The Web of Science Core Collection database was used for this quantitative analysis. The search formula was TS = (“Platelet-rich plasma” treatment OR therapy OR treating OR therapeutic OR cure OR healing). The inclusion period spanned from January 1, 2000, to October 1, 2022, ensuring the capture of recent and relevant research. The search, conducted on October 29, 2022, was limited to English-language articles to maintain language consistency and avoid barriers. The results were downloaded in Tab Delimited and Plain Text formats for further analysis. Figure 1 illustrates the literature screening process, which included an initial search based on the defined formula, exclusion of duplicates, application of a language filter (English), and the final selection of eligible documents.

### 2.2. Data and visual analysis

Centrality, a network metric used in CiteSpace, measures the importance of nodes, visually represented by purple circles. Total link strength in VOSviewer quantifies the strength of connections, with higher total link strength indicating stronger relationships. The H-index reflects a researcher’s productivity and impact, with higher values indicating greater influence. The Journal Impact Factor measures journal-level impact based on citations in the Web of Science Core Collection, and Journal Citation Reports assigns quartiles to journals based on their impact factor scores.

We used VOSviewer 1.6.18 for mapping networks of countries, institutions, authors, keywords, and journals, revealing collaboration patterns and research hotspots.^[15]^ CiteSpace 6.1 analyzed national distributions, dual map overlays of journals, keyword timelines, and subject areas.^[16]^ Bibliometrix (R-Studio’s R-Tool) detailed authors, references, and keywords.^[17]^ TBtools 1.1043 created keyword heat maps.^[18]^ SCImago Graphica Beta 1.0.26 mapped the global cooperation network by country or region.^[19]^ Origin 2021b represented publication dynamics, showing trends and changes in publication output over time for countries and journals.

## 3. Results

### 3.1. Trends in annual publications and citations

**Figure 2.**
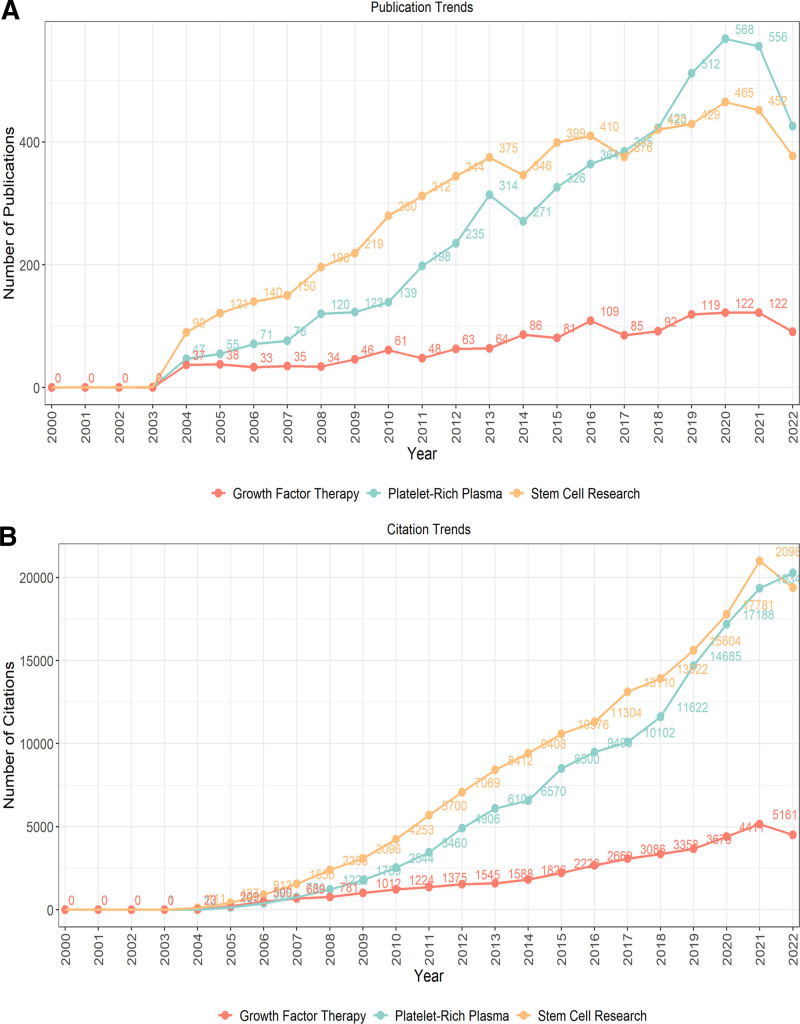
Trends in PRP publications and citations compared to other therapies (2000–2022). (A) Annual number of publications for PRP, stem cell research, and growth factor therapy. (B) Annual number of citations for PRP, stem cell research, and growth factor therapy.

Figure 2 showcases the notable growth in PRP publications and citations from 2000 to 2022, alongside a comparative analysis with stem cell research and growth factor therapy.

Figure 2A demonstrates a notable increase in PRP-related publications, with PRP surpassing growth factor therapy by 2004 and exceeding stem cell research by 2017. This indicates the growing prominence of PRP in the field of regenerative medicine. The sharp rise in PRP publications from 2018 further emphasizes its increasing influence and adoption in clinical and academic settings. Figure 2B reveals PRP’s extensive academic and clinical impact, with citations peaking in 2022. This surge aligns with PRP’s enhanced application in clinical settings, validated by a robust increase in citations, eventually exceeding those of stem cell research by the end of the analyzed period.

These observations highlight the expanding recognition and utilization of PRP within the scientific and medical communities, positioning it as a significant player in regenerative therapies alongside established fields like stem cell research (Table S1, Supplemental Digital Content, http://links.lww.com/MD/N948). The increase in publications and citations highlights both the historical context and the current advancements, providing a comprehensive overview of PRP research over the past 2 decades.

### 3.2. Geographic distribution of research

**Figure 3.**
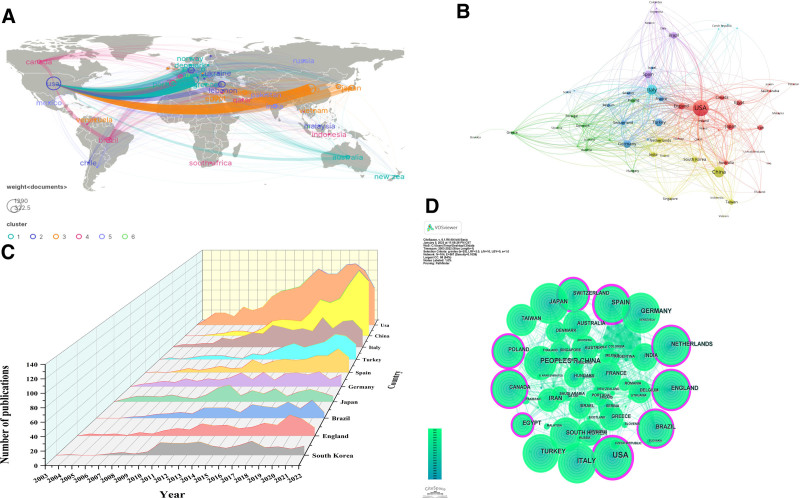
Global distribution and collaborative dynamics in PRP research. (A) Geographic distribution of PRP research, highlighting concentration in the northern hemisphere. (B) Network visualization of country collaborations, with node size representing publication volume and different colors indicating regions. (C) Annual publication trends from the top 10 countries over 22 years, showing growth in PRP research. (D) CiteSpace visualization of the USA’s central role in global PRP research.

PRP research demonstrates a global reach, spanning 105 countries predominantly in the northern hemisphere, with North America, Europe, and Asia being the primary contributors (Fig. 3A). This distribution highlights extensive international collaboration. The United States emerges as a leader in PRP research, with outputs of 1290 articles and 40,789 citations (Fig. 3B and C). The CiteSpace visualization (Fig. 3D) emphasizes the USA’s central role, showcasing its influential network and collaborations.

### 3.3. Institutional contributions

**Figure 4.**
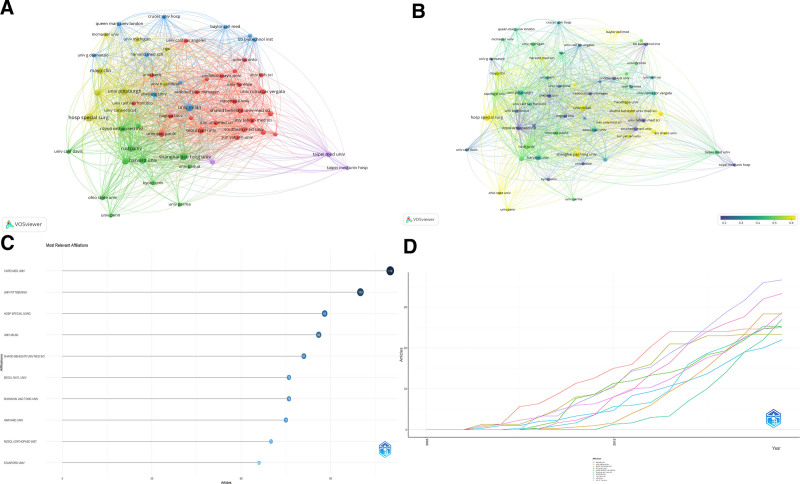
Institutional dynamics in PRP research. (A) Collaborative networks: maps inter-institutional collaborations, showing publication frequency and research group affiliations by node size and color. (B) Research activity heat map: highlights recent research intensity compared to historical performance, calculated by publication metrics over the past 5 years. (C) Leading institutions: identifies institutions pivotal in PRP research, emphasizing their impact and contributions. (D) Publication trends: charts the publication output of prominent institutions over time, revealing trends in PRP research focus.

Table S2, Supplemental Digital Content, http://links.lww.com/MD/N949 lists the top institutions by publication volume in PRP research, highlighting the Hospital for Special Surgery and Harvard University as leaders, primarily from the United States. Table S3, Supplemental Digital Content, http://links.lww.com/MD/N950 details influential institutions by citation impact, with notable mentions of The Rizzoli Orthopaedics Institute and Cornell University. Figure 4A shows a network of 57 institutions, illustrating strong collaborative ties through clustering. Figure 4B presents a heat map of the last 5 years, pinpointing emerging leaders like the Hospital for Special Surgery and the University of Milan, while noting a decrease in contributions from institutions like Nagoya University. Figure 4C highlights the most influential institutions in PRP research, while Figure 4D illustrates the publication trends over time for each of these leading institutions.

### 3.4. Distribution of authors

**Figure 5.**
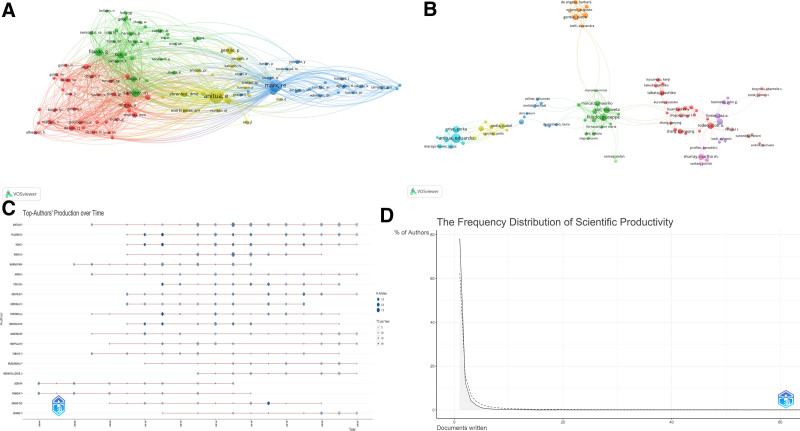
Authorship analysis in PRP research. (A) Co-citation network: maps inter-author citation links, showing thematic clusters. (B) Collaboration patterns: outlines major collaborative networks and key contributors. (C) Top-authors’ production over time: reflects the publication output of leading authors over time. (D) Author productivity via Lotka law: analyzes publication frequency distribution, highlighting prolific contributors and one-time publishers.

Author co-citation analysis, involving a network of 23,894 authors, highlights scholarly connections and thematic overlaps by tracking citation frequencies. Tables S4, Supplemental Digital Content, http://links.lww.com/MD/N951 and S5, Supplemental Digital Content, http://links.lww.com/MD/N952 showcase leading authors by publication and co-citation frequencies, with Anitua Eduardo standing out for his substantial impact. Figure 5A and B visualize co-citation and collaboration networks, emphasizing central figures like Filardo Giuseppe. Figure 5C tracks publication trends over time, while Figure 5D applies Lotka Law, which states that the number of authors publishing n papers is approximately 1/n^2^ of those publishing one paper, showing that about 78.2% of contributors are single-publication authors, reflecting the diverse contributor base in PRP research.

### 3.5. Distribution of the journals

**Figure 6.**
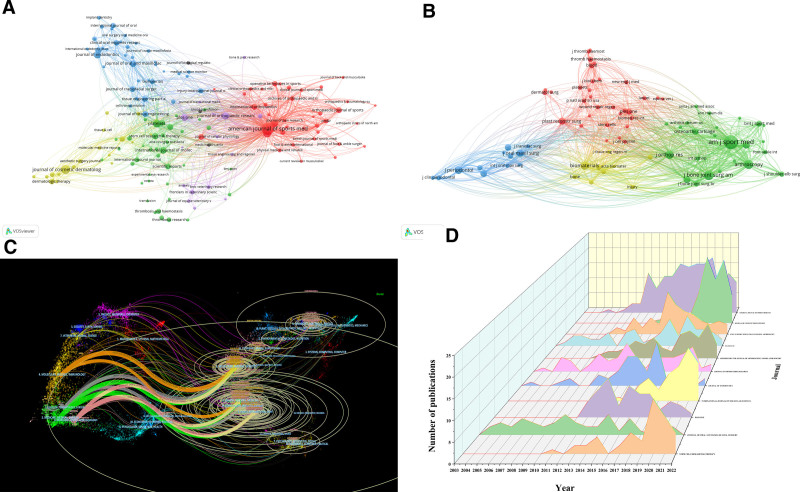
Journal analysis in PRP research. (A) Network visualization in VOSviewer: Groups journals by thematic research areas, with node size indicating publication frequency. (B) Co-citation analysis in VOSviewer: reveals patterns of influence and collaboration among 77 journals. (C) Dual-map overlay: shows interdisciplinary citation flows between citing and cited journals. (D) Publication trends: tracks changing publication activity of leading journals in PRP research.

An analysis of 1128 journals reveals trends in PRP research, with The American Journal of Sports Medicine leading in publications and co-citations, highlighting its central role in sports medicine (Tables S6, Supplemental Digital Content, http://links.lww.com/MD/N953 and S7, Supplemental Digital Content, http://links.lww.com/MD/N954). Figure 6A and B present network visualizations of journals and their co-citations, respectively, categorizing them into thematic clusters: such as orthopedics and sports medicine in red or dentistry and implants in blue. Figure 6C uses a dual map overlay to show interdisciplinary citations across fields like dentistry, surgery, and sports medicine, highlighting their connections to genetics, chemistry, and rehabilitation. Figure 6D displays the increasing trend in PRP publications by leading journals, highlighting the growing research interest.

### 3.6. Distribution of references

**Figure 7.**
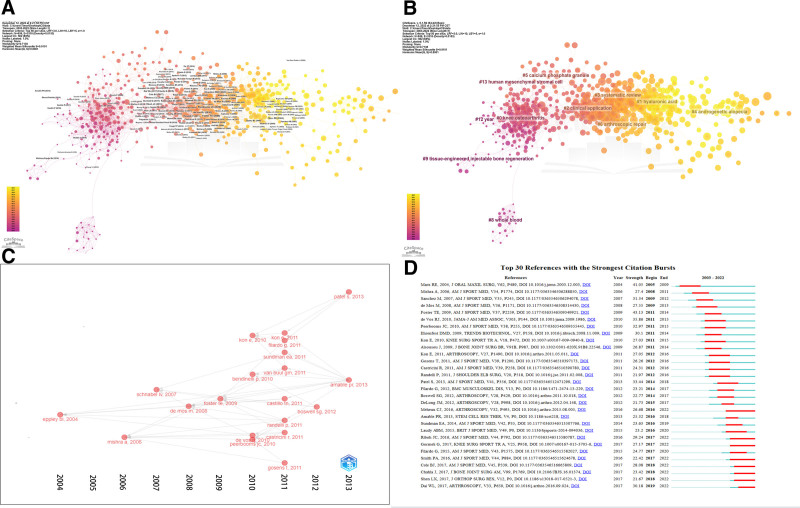
Reference analysis in PRP research. (A) Network analysis in CiteSpace: highlights co-citation relationships, revealing key clusters and topics. (B) Clustering analysis in CiteSpace: organizes references into thematic clusters like knee osteoarthritis and hyaluronic acid. (C) Historiograph: maps the development and influence of seminal works overtime. (D) Citation bursts: identifies top 30 references with increases in citations, indicating shifts in research impact.

Tables S8, Supplemental Digital Content, http://links.lww.com/MD/N955 and S9, Supplemental Digital Content, http://links.lww.com/MD/N956 highlight the top articles in PRP research. Marx Robert E. and Foster Timothy E. are notable for their influential studies on PRP’s clinical applications. The network analysis of the co-cited references can be seen in Figure 7A and Figure S1, Supplemental Digital Content, http://links.lww.com/MD/N946. Based on the correlation between articles, articles were divided into 11 clusters and represented by different colors (Fig. 7B). Figure 7C’s historiograph traces the connections among seminal articles, with Eppley Barry 2004 work as a cornerstone. Figure 7D identifies articles with citation bursts, indicating evolving research priorities.

### 3.7. Distribution of keywords

**Figure 8.**
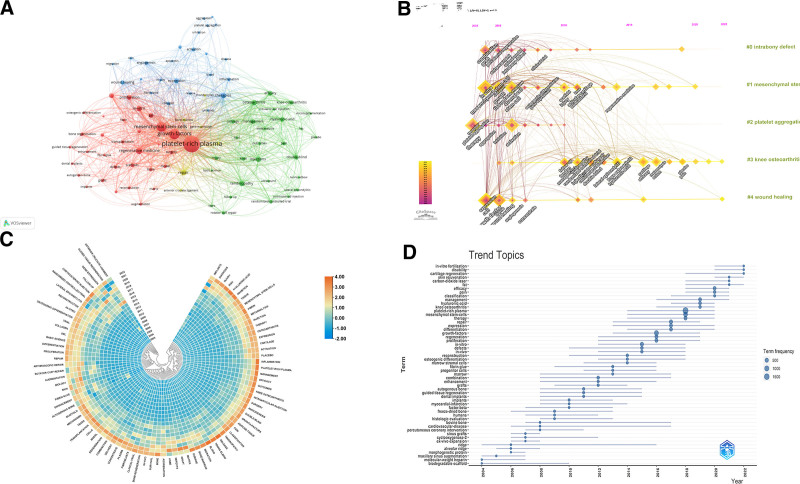
Keyword analysis in PRP research. (A) Co-occurrence in VOSviewer: displays keywords appearing more than 65 times, with node size indicating frequency and color denoting clusters. (B) Timeline in CiteSpace: shows keyword clusters over time, with lines representing co-citation relationships. (C) Heat map: highlights keywords by yearly citation proportion, indicating research focus areas. (D) Trending topics: identifies emerging keywords, indicating shifts in PRP research focus.

Keyword analysis from 5167 articles identified 13,802 unique terms, with “Platelet-Rich Plasma,” “Growth Factors,” and “Mesenchymal Stem Cells” as the most prevalent. Figure 8A categorizes keywords into research themes, such as regenerative medicine (red cluster) and orthopedic conditions (green). Figure 8B maps the evolution of keywords from “Combination” and “Graft” to “Stromal Vascular Fraction” and “Fibrin.” Figure 8C highlights shifts in keyword popularity, noting increases for “Inflammation” and “Bone Regeneration.” Figure 8D identifies emerging topics, including “In Vitro Fertilization” and ‘Pain.’ Table S10, Supplemental Digital Content, http://links.lww.com/MD/N957 further details the top 20 keywords by frequency, providing deeper insight into trending research topics.

### 3.8. Distribution of subject area

CiteSpace’s analysis of subject areas in PRP literature delineates the disciplines most engaged with PRP research, as detailed in Figure S2, Supplemental Digital Content, http://links.lww.com/MD/N947. Prominent subjects include “Dentistry, Oral Surgery & Medicine,” “Sport Sciences,” “Orthopedics,” and “Dermatology.” The purple rings around these subjects signify their substantial influence and centrality in PRP research, highlighting these disciplines as key fields of study.

## 4. Discussion

### 4.1. Status of PRP research

#### 4.1.1. PRP research overview based on bibliometric analysis

Our bibliometric analysis of PRP research from 2000 to 2022 draws on 5167 articles from 5562 institutions and 23,894 authors, highlighting the field’s evolution and broad application. The increasing volume of publications and citations, peaking in 2020 and 2021, underscores a global effort led by the United States. Key institutions like the Hospital for Special Surgery, Harvard University, and the University of Milan have been instrumental in advancing PRP research.

Influential researchers such as Anitua Eduardo and Marx Robert E have shaped PRP research, setting standards for scholarly rigor and innovation. The American Journal of Sports Medicine exemplifies this, serving as a focal point for cutting-edge studies. Keyword analysis reveals an expansion from traditional applications in orthopedics and dentistry to diverse fields including cosmetic dermatology and cardiovascular disease. Recent keywords like “Management” and “Efficacy” emphasize the need for standardized PRP protocols to enhance safety and effectiveness. Emerging keywords such as “Plasma Gel” and “Hydrogel” indicate innovative delivery methods being explored. The integration of PRP with advanced technologies like “Carbon-Dioxide Laser” and “Micro needling” points to new multi-modal treatment strategies. Moreover, the increasing focus on “Platelet-Derived Exosomes” and “Microvesicles” suggests expanding research beyond traditional PRP applications, exploring a wider spectrum of applications and studies in platelet therapies.

#### 4.1.2. Current hotspots in PRP application

From the co-occurrence network diagram and timeline graphic analysis derived from our bibliometric study, recent themes and keywords such as “Tendinopathy,” “Tennis Elbow,” “Osteoarthritis,” and “Bone Regeneration” stand out. This pinpoints the predominant research directions, mainly focusing on Orthopedics and Sports Medicine. Initially, the realm of orthopedics and sports medicine observed pronounced benefits. Conditions such as tennis elbow, rotator cuff injuries, patellar tendonitis, and muscle strains have seen improvements with PRP treatment, evidenced by accelerated healing pathways and symptom alleviation. Empirical studies support PRP’s role in promoting bone healing, chondrocyte proliferation, cartilage preservation, and inflammatory modulation. This scientific validation has fueled the growing interest and extensive research in this area, making it a hotspot for PRP applications.^[20–25]^ The onward journey of PRP soon intersected with dentistry and oral medicine.

Recent bibliometric analysis highlights key themes such as “Dental Implants,” “Periodontal Regeneration,” and “Tooth Extraction,” emphasizing the impact of PRP in these areas. In Dentistry, PRP has optimized various procedures including tooth extractions, periodontal treatments, and dental implant surgeries. It has shown efficacy in promoting soft and hard tissue regeneration, reducing postoperative complications, and enhancing overall treatment outcomes. Empirical studies demonstrate PRP’s effectiveness in stimulating periodontal tissue regeneration, improving indicators associated with generalized aggressive periodontitis, mitigating gum recession, and enhancing dentition stability. These findings have driven the increasing research focus on PRP in dentistry and oral medicine, establishing it as a major hotspot.^[26–31]^

### 4.2. Future directions in PRP research

#### 4.2.1. Potential areas in PRP application

**Figure 9.**
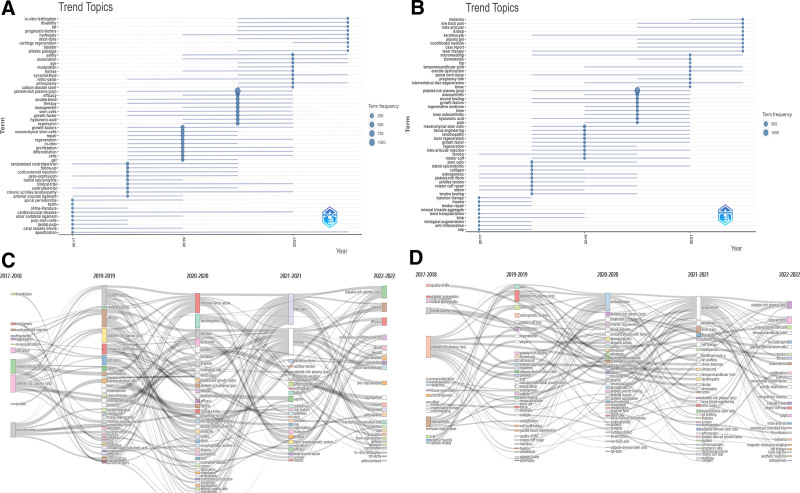
Current hotspots and future directions in PRP research (2017–2022). (A) trending topics in PRP 2017 to 2022, by author’s keywords analysis. (B) Trending topics in PRP 2017 to 2022, by author’s keywords plus analysis. (C) Thematic evolution in PRP 2017 to 2022, by author’s keywords analysis. (D) Thematic evolution in PRP 2017 to 2022, by author’s keywords plus analysis.

Based on an extensive analysis spanning from 2017 to 2022, our thematic exploration of PRP applications delineates significant advancements across various medical disciplines, as evidenced in Figure 9A–D.

In Dermatology, PRP has been substantiated as an effective treatment for hair loss, acne vulgaris, and striae distensae, where it improves skin texture and follicle vitality.^[32–37]^ Medical Aesthetics employs PRP for skin rejuvenation, significantly reducing wrinkles and treating hyperpigmentation disorders such as melasma and vitiligo, with synergistic effects noted when combined with microneedling.^[38–44]^ Reproductive Medicine highlights PRP’s role in enhancing sperm quality and facilitating endometrial regeneration, thus improving therapeutic outcomes in both male and female fertility issues.^[45–50]^ In Ophthalmology, PRP supports the healing of dry eyes and corneal ulcers, accelerating recovery and improving visual function.^[51–55]^ Urology applications of PRP show efficacy in managing bladder disorders like interstitial cystitis and in treatments for pelvic organ prolapse and urinary incontinence, enhancing patient quality of life.^[56–60]^ Nerve Disorders benefit from PRP’s ability to promote nerve regeneration, particularly in peripheral nerve injuries, with emerging evidence supporting its use in central nerve recovery.^[61–64]^ In Otolaryngology, PRP is utilized for rapid healing of tympanic membrane perforations and for treating vocal cord lesions, improving both structural integrity and function.^[65–71]^ Although less prevalent in Cardiovascular Medicine, PRP assists in managing refractory angina and postoperative recovery in cardiac surgeries, demonstrating its broad therapeutic potential.^[72–76]^ This multifaceted utility of PRP across diverse medical fields underscores its integral role in regenerative medicine, offering promising avenues for future clinical applications and research.

**Figure 10.**
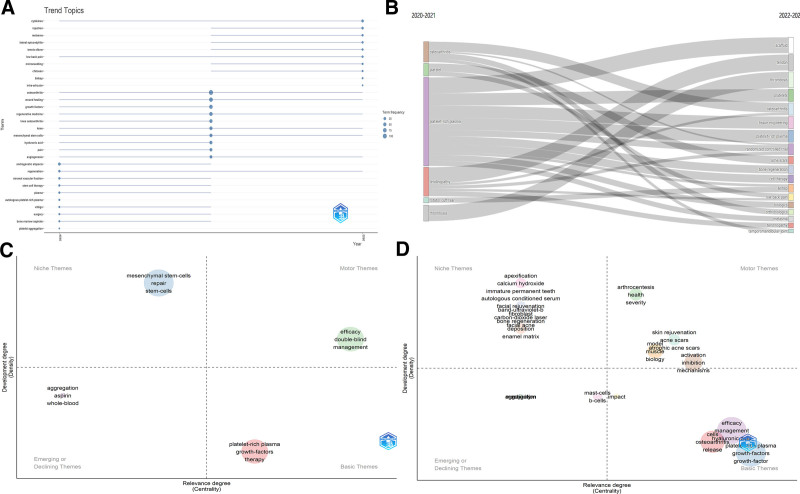
Current hotspots and future directions in PRP research (2020–2022). (A) Trend topics: highlights dominant keywords and central themes. (B) Thematic evolution (2020–2022): visualizes shifts in research themes and technological advancements. (C) Initial phase (2020–2021): details early thematic changes and innovations. (D) Latter phase (2021–2022): examines ongoing adjustments and new methodologies as the field evolves.

To comprehensively understand the evolving landscape of PRP applications, we analyzed recent trends and thematic evolution from 2020 to 2022. Figure 10A highlights trending keywords, showcasing established areas such as “Platelet-Rich Plasma,” “Osteoarthritis,” and “Growth Factors,” alongside emerging fields like “Microneedling,” “Melasma,” “Tennis Elbow,” and “Vitiligo.” This indicates PRP’s expanding therapeutic scope into dermatology, cosmetic procedures, and chronic pain management. The thematic evolution presented in Figure 10B illustrates a shift from core topics such as “Osteoarthritis” and “Platelet-Rich Plasma” towards specialized and interdisciplinary themes like “Tissue Engineering,” “Scaffold,” and “Randomized Controlled Trials.” This transition reflects the maturation of PRP research from foundational studies to application-driven investigations, emphasizing its integration into clinical practice.

Figure 10C and D further dissect the thematic evolution by dividing the period into 2 phases: 2020 to 2021 and 2021 to 2022. During the initial phase (2020–2021), research predominantly focused on orthopedic and musculoskeletal conditions, with prominent keywords such as “Osteoarthritis,” “Tendinopathy,” and “Rotator Cuff Tear.” This aligns with PRP’s traditional applications in enhancing musculoskeletal healing. However, the latter phase (2021–2022) reveals a diversification of research interests, with an increasing focus on “Acne Scars,” “Melasma,” and “Low Back Pain,” indicating a shift towards aesthetic and chronic pain management applications.^[77,78]^ Strategic diagrams in Figure 10C and D categorize themes based on their density and centrality, offering insights into niche and motor themes within PRP research. “Mesenchymal Stem Cells” and “Stem Cell Therapy” emerge as niche themes with high potential for development, while “Efficacy,” “Double-Blind,” and “Management” are identified as motor themes, indicating well-established areas driving the field forward. These insights suggest a growing interest in combining PRP with advanced regenerative techniques, such as stem cell therapy, to enhance treatment outcomes.

#### 4.2.2. Advancements in PRP delivery pathways

Innovative delivery mechanisms for PRP enhance its clinical applicability and efficacy by addressing limitations of traditional methods. Recent advancements enable precise, controlled, and sustained release of PRP components, broadening its applications across various medical fields.^[75,79–84]^

The effectiveness of PRP treatments relies not only on the biological properties of the plasma but also on the precision of its delivery to target tissues. While direct injection promotes tissue regeneration, it faces challenges in ensuring even distribution within complex tissue structures. Gel carriers, which mix PRP with gel materials, allow for slow release and enhanced bioavailability, though their preparation can be complex.^[85]^ PRP sprays offer quick and even distribution over large areas but may lack depth in tissue penetration.^[79,86]^

Advanced delivery methods, such as microneedling and nanoparticle-based systems, improve patient experiences by reducing discomfort and accelerating recovery times.^[87]^ Microneedling creates microchannels in tissue, facilitating deeper PRP delivery with minimal invasiveness. Nanoparticle-based systems offer sustained release, reducing the need for multiple treatments and improving patient compliance. Ultrasound-guided injections enhance the precision of PRP treatments by enabling real-time visualization and accurate targeting of treatment areas, reducing invasiveness.

Exploring diverse delivery methods reveals potential new therapeutic areas for PRP application. Traditional scaffolds provide continuous PRP release and structural support for tissue repair but face biocompatibility and cost challenges.^[88]^ 3D-printed scaffolds, customizable to specific anatomical needs, enhance precision and efficiency in PRP delivery, particularly in orthopedics and dentistry.^[89]^

Despite these advantages, innovative PRP delivery mechanisms face challenges like regulatory hurdles, high costs, and the need for specialized training. Ensuring the long-term safety and biocompatibility of new materials is crucial. Future research should focus on optimizing delivery systems for clinical efficacy and safety, exploring biodegradable scaffolds, localized release systems, and bioengineered vehicles. Comparative studies of traditional versus innovative methods can guide clinical decisions, enhancing PRP’s therapeutic potential and improving patient outcomes across various medical fields.

#### 4.2.3. Ensuring the safety and ethical considerations of PRP treatments

Ensuring the safety and addressing the ethical considerations of PRP treatments is essential for maintaining patient trust and upholding medical practice integrity.

Although PRP, being an autologous process, reduces immunological reactions compared to allogeneic treatments, it is not without risks. Potential side effects such as pain, swelling, discomfort, and allergic reactions to substances used in PRP preparation must be clearly communicated to patients during the informed consent process.^[90–95]^ Patients with clotting disorders or those on anticoagulant therapy require special attention due to an increased risk of bleeding and bruising. Healthcare providers must evaluate whether the therapeutic benefits of PRP outweigh these risks. Long-term safety and the immunogenic potential of PRP, including the risk of autoimmune or hypersensitivity reactions, remain underexplored and require further research. Standardized protocols are critical for enhancing safety and ensuring consistent outcomes, defining optimal conditions for blood processing, and specifying PRP formulations and administration techniques tailored to therapeutic needs.

Ethical considerations are paramount in PRP treatments. Patients must be fully informed about the PRP treatment process, including procedural steps, potential discomfort, recovery expectations, and costs, which are often not covered by insurance. Comprehensive product information and clinical evidence supporting PRP use for specific conditions should be provided. Healthcare providers must present the effectiveness and risks of PRP treatments transparently, offering unbiased information and prioritizing patient welfare over commercial interests. Ensuring patient privacy and data protection, adhering to relevant privacy laws, is essential. Equitable access to PRP treatments should be guaranteed based on clinical needs, not economic capabilities. Disclosing any potential conflicts of interest is crucial to maintaining trust and integrity. Decisions regarding patient care must be driven by clinical needs and supported by scientific evidence. Ongoing ethical education for medical practitioners is necessary to ensure they are well-informed about the latest ethical guidelines.

By addressing these ethical considerations, PRP treatments can be administered safely and respectfully, enhancing the credibility and reliability of PRP as a therapeutic option while upholding the highest standards of medical ethics.

#### 4.2.4. Comparative analysis of PRP and other regenerative therapies

To effectively understand the place of PRP within the domain of regenerative medicine, it is crucial to conduct a comparative analysis with other key regenerative therapies.

Stem cell therapy utilizes multipotent or pluripotent stem cells for tissue regeneration, addressing conditions from degenerative diseases to injuries.^[96]^ While it offers direct tissue regeneration capabilities, it is costly, complex, and subject to strict regulations due to ethical concerns and tumorigenesis risks. Compared to PRP, stem cell therapy involves higher risks and costs, making PRP a safer and more cost-effective option where growth factors alone can induce effective tissue regeneration.

Growth factor therapy administers specific growth factors to stimulate healing, providing consistent and predictable outcomes.^[97]^ Unlike PRP’s natural mixture of growth factors, this therapy uses standardized doses of individual factors. While growth factor therapy lacks PRP’s synergistic benefits, PRP’s multifactorial composition is more suitable for broader applications requiring combined effects.

Exosome therapy uses platelet-derived exosomes for targeted delivery of bioactive molecules, enhancing tissue repair and immune modulation.^[98]^ Though exosome therapy offers high specificity and reduced immunogenicity, it is technically challenging and costly. PRP, while lacking precise targeting, is simpler and more accessible.

Platelet-derived microvesicles (PMVs) and platelet lysate represent advancements within PRP-derived therapies.^[99,100]^ PMVs, rich in bioactive molecules, play roles in hemostasis and inflammation regulation, while platelet lysate releases growth factors valuable for tissue engineering and wound healing. Both PMVs and PL, being autologous, minimize immune reactions but lack PRP’s broad applicability and ease of preparation.

#### 4.2.5. Standardizing, personalization, and optimization of PRP protocols

The widespread adoption of PRP across various medical specialties necessitates robust standardization to ensure consistent, safe, and effective outcomes. Standardizing PRP preparation involves uniform blood collection and centrifugation techniques while accommodating individual biological variability. Protocols must adapt to unique platelet concentrations and bioactive factors in each patient’s blood. Factors such as anticoagulants and processing conditions influence PRP quality. Advanced automation in PRP preparation meticulously controls processing parameters, reducing variability and enhancing reproducibility.^[101]^ Using standardized equipment, such as specific centrifuge models with defined settings, ensures consistency across clinical settings. Medical-grade reagents and materials are necessary to maintain PRP’s safety and effectiveness. Regular testing of platelet concentration, leukocyte and erythrocyte admixture, and microbial contamination is critical for maintaining PRP quality. Despite controversy over the retention of red and white blood cells in PRP, standardized testing and preparation protocols help manage this variability.

Optimizing PRP involves several factors, with platelet concentration being pivotal. While high platelet concentrations can enhance tissue healing and regeneration, lower concentrations can also be effective. Studies have shown that higher platelet concentrations do not necessarily result in better outcomes, highlighting the variability in PRP preparation methods.^[102–107]^ The effectiveness of PRP is also influenced by growth factors, cytokines, and extracellular matrix proteins. Therefore, optimal platelet concentration may vary depending on tissue repair requirements. Establishing standardized concentrations tailored to specific indications is crucial for enhancing clinical outcomes.

Standardizing PRP application is essential for consistent, safe, and effective outcomes. This involves creating precise guidelines for administration methods and routes, including injection, topical application, and implantation. Each method should be tailored to the patient’s condition, targeted treatment area, and therapeutic goals. Standardized protocols should define the optimal dosage, frequency, and timing of PRP applications. Appropriate injection site, depth, and angle are critical for effective delivery. Imaging technologies like ultrasound or MRI can enhance precision and therapeutic efficacy by providing real-time feedback. Integrating PRP with other therapies, such as stem cells, hyaluronic acid, or physical therapy, can enhance treatment outcomes. Guidelines for combination therapies should determine the optimal sequence and timing for administering PRP alongside other treatments.

Standardizing the evaluation, ethical, regulatory and storage aspects of PRP treatments is crucial. This includes establishing uniform clinical outcome measures, such as pain scores and functional improvement assessments, and implementing robust biological assessments like biomarker analysis and immune response evaluations. Compliance with regulatory standards set by bodies such as the Food and Drug Administration or European Medicines Agency is essential for maintaining patient trust and legal integrity. Detailed informed consent processes must ensure patients are fully aware of the potential risks and benefits of PRP therapy. Guidelines for integrating PRP with other therapeutic modalities should specify the optimal sequence and timing for administering PRP alongside other treatments. Standardizing PRP clinical trials’ design, conduct, and reporting will improve the quality and reliability of study results, facilitating evidence-based decision-making and advancing regenerative medicine. Storage methods, including temperature and time, significantly impact PRP efficacy, with no consensus on optimal conditions. Proper transport is also crucial to maintain PRP quality. Standardizing storage and transport protocols ensures PRP stability, efficacy, and comparability of clinical outcomes.^[108–111]^

Personalization of PRP therapy signifies a meticulous adaptation of treatment protocols to cater to the unique physiological and pathological attributes of individual patients, encompassing aspects such as age, gender, race, and the specific ailment being addressed. This comprehensive tailoring extends into the realms of defining PRP composition, particularly the concentration of platelets, leukocytes, and growth factors, and discerning administration routes, all aimed at amplifying therapeutic efficacy while concurrently mitigating any potential adverse effects.^[112]^ It necessitates a nuanced approach, adapting not only to the individual characteristics and responses of the patient but also to the particularities of their pathology, ensuring an ideal alignment between the treatment modality and the pathological target. On the other hand, the optimization of PRP encapsulates an ongoing discourse regarding the ideal platelet concentrations and the concurrent roles of other PRP constituents such as cytokines and growth factors in engendering therapeutic effects. Acknowledging that effectiveness can be witnessed across a spectrum of platelet concentrations, optimization endeavors to delineate standardized platelet concentrations, which are both potent and safe, and are meticulously tailored to specific indications and pathological conditions. Herein, the aim is not merely to establish a “one-size-fits-all” criterion but to devise a flexible, yet evidence-based framework that allows for variability while ensuring consistency in therapeutic outcomes. The convergence of personalization and optimization, therefore, resides in establishing a synergistic balance that both respects individual patient attributes and adheres to an optimized, evidence-based protocol, ensuring that PRP therapy is not only maximally effective but also robustly safe across a diverse array of clinical applications within regenerative medicine. While standardization seeks to establish consistency and reproducibility in PRP therapy, it must be judiciously balanced with personalization and optimization efforts to ensure that treatments are optimally tailored to individual patient needs.

The manifold potential of PRP unfolds a rich tapestry of therapeutic vistas, weaving together promising opportunities and a nexus of controversies and ethical dilemmas in regenerative medicine. The wide-ranging clinical applications of PRP, which permeate various medical disciplines, demand a delicate equilibrium between the imperatives of standardization and the finesse of personalized approaches, ensuring ethical and economic accessibility across diverse patient demographics. Amidst the fervent discourse regarding optimal platelet concentration and the binary of leukocyte presence within PRP, medical professionals are tasked with unraveling the intricate correlations between platelet concentration, therapeutic efficacy, and potential immunological ramifications, navigating through the therapeutic landscapes of leukocyte-rich and leukocyte-poor PRP. As the horizon of PRP expands, marrying innovative strategies and venturing into unexplored therapeutic territories, the amalgamation of therapeutic modalities and exploration of innovative delivery platforms such as hydrogels and microneedling, pave the way towards augmenting therapeutic outcomes and devising pioneering treatment paradigms. The transition of PRP from the experimental stage to a staple in clinical practice necessitates the formulation of stringent safety protocols, comprehensive risk management frameworks, and sturdy patient monitoring mechanisms, safeguarding patient wellbeing whilst optimizing therapeutic efficacy. Thus, the forward trajectory of PRP is charted, steered by perpetual exploration, rigorous critical appraisal, and systematic implementation, propelling its evolution from experimental endeavor to a standardized, safe, and efficacious practice, resonating across an array of medical disciplines.

## 5. Conclusion

Our bibliometric analysis of PRP research from 2000 to 2022 reveals its expanding applications beyond orthopedics into dermatology, pain management, and cosmetic procedures. Despite its versatility, there is a critical need for standardized preparation and application protocols to ensure consistency in clinical outcomes. Future research should focus on standardizing PRP protocols, personalizing treatments to enhance efficacy, and exploring innovative delivery methods. Comprehensive safety assessments and adherence to ethical standards are essential. Additionally, comparative studies with other regenerative therapies are necessary to identify potential synergistic effects.

By addressing these areas, PRP’s therapeutic potential can be fully harnessed, establishing it as a cornerstone of regenerative medicine and offering versatile solutions to a wide array of clinical challenges.

## 6. Limitations

The limitations of this study warrant consideration to ensure a balanced interpretation of the findings. Primarily, our analysis was limited to English-language publications, potentially sidelining key insights from non-English research and introducing a linguistic bias. Additionally, while we aimed to address PRP-related controversies, the exploration of optimal platelet concentrations might not be exhaustive. Our data sourcing was predominantly from the Web of Science Core Collection, which may exclude studies indexed in other databases. The findings are also temporally bound, capturing only publications available until October 1, 2022, thus any subsequent developments in the field remain outside the scope of this analysis. The choice of analytical tools was determined by their availability and relevance during our research phase, which may have inherent constraints. Emerging tools or methodologies could potentially provide nuanced or slightly varied insights from the same dataset. The study primarily reflects recent trends in PRP research, introducing potential temporal bias. Future studies should balance different periods and employ longitudinal methods to capture a more comprehensive landscape.

## Acknowledgments

We would like to express our gratitude for the free use of the various software tools that made this research possible, including CiteSpace, VOSviewer, Bibliometrix, TBtools, SCImago Graphica, Origin, and Excel. Their accessibility and functionality were instrumental in the completion of our bibliometric analysis.

## Author contributions

**Conceptualization:** Kai Du, Shu-Ming Li.

**Supervision:** Ren Guo, Shu-Ming Li.

**Writing – original draft:** Kai Du, Ao Li.

**Writing – review & editing:** Chen-Yu Zhang.

## Supplementary Material

**Figure:**
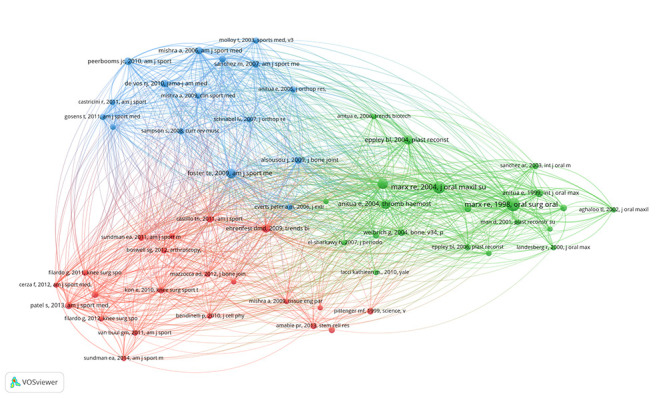


**Figure:**