# Supplementary material for: Platelet-rich plasma: A bibliometric and visual analysis from 2000 to 2022
Source: Medicine (Baltimore). 2024 Nov 15;103(46):e40530. doi: 10.1097/MD.0000000000040530 (PMC11575995; doi:10.1097/MD.0000000000040530)
Supplement: Supplementary file 1 [file medi-103-e40530-s001.docx]

Platelet-Rich Plasma：A Bibliometric and Visual Analysis from 2000 to 2022

Supplementary Tables

**Supplementary Table 1 Top 10 countries or regions in terms of the number of publications**

| Rank | Countries or regions | Publications | Citations | Centrality |
| --- | --- | --- | --- | --- |
| 1 | USA | 1290 | 40789 | 0.28 |
| 2 | China | 614 | 11642 | 0.06 |
| 3 | Italy | 528 | 19003 | 0.06 |
| 4 | Turkey | 317 | 4227 | 0.04 |
| 5 | Spain | 299 | 6227 | 0.21 |
| 6 | Germany | 282 | 8896 | 0.08 |
| 7 | Japan | 247 | 7926 | 0.04 |
| 8 | Brazil | 224 | 3747 | 0.10 |
| 9 | England | 216 | 6200 | 0.14 |
| 10 | South Korea | 187 | 6394 | 0.03 |
