# Supplementary material for: Platelet-rich plasma: A bibliometric and visual analysis from 2000 to 2022
Source: Medicine (Baltimore). 2024 Nov 15;103(46):e40530. doi: 10.1097/MD.0000000000040530 (PMC11575995; doi:10.1097/MD.0000000000040530)
Supplement: Supplementary file 2 [file medi-103-e40530-s002.docx]

Platelet-Rich Plasma：A Bibliometric and Visual Analysis from 2000 to 2022

Supplementary Tables

**Supplementary Table 2 Top 10 institutions in terms of the number of publications**

| Rank | Institutions | Records | | Percentage (%) | Citations | Centrality |
| --- | --- | --- | --- | --- | --- | --- |
| 1 | Hospital For Special Surgery | | 79 | 1.5 | 2556 | 0.12 |
| 2 | Harvard University | | 75 | 1.4 | 2614 | 0.10 |
| 3 | University Of Milan | | 64 | 1.2 | 2418 | 0.07 |
| 4 | Shanghai Jiao Tong University | | 62 | 1.2 | 1589 | 0.02 |
| 5 | University Of Pittsburgh | | 55 | 1.1 | 2049 | 0.04 |
| 6 | Rush University | | 50 | 1.0 | 2271 | 0.06 |
| 7 | Cornell University | | 48 | 0.9 | 3181 | 0.02 |
| 8 | Mayo Clinic | | 48 | 0.9 | 1134 | 0.05 |
| 9 | Stanford University | | 48 | 0.9 | 3084 | 0.06 |
| 10 | Seoul National University Snu | | 46 | 0.9 | 1921 | 0.01 |
