# Supplementary material for: Platelet-rich plasma: A bibliometric and visual analysis from 2000 to 2022
Source: Medicine (Baltimore). 2024 Nov 15;103(46):e40530. doi: 10.1097/MD.0000000000040530 (PMC11575995; doi:10.1097/MD.0000000000040530)
Supplement: Supplementary file 3 [file medi-103-e40530-s003.docx]

Platelet-Rich Plasma：A Bibliometric and Visual Analysis from 2000 to 2022

Supplementary Tables

**Supplementary Table 3 Top 10 most cited institutions**

| Rank | Institutions | Records | Percentage (%) | Citations | Centrality |
| --- | --- | --- | --- | --- | --- |
| 1 | The Rizzoli Orthopaedics Institute | 37 | 0.7 | 3283 | 0.04 |
| 2 | Cornell University | 48 | 0.9 | 3181 | 0.02 |
| 3 | Stanford University | 48 | 0.9 | 3084 | 0.06 |
| 4 | Harvard University | 75 | 1.4 | 2614 | 0.10 |
| 5 | Hospital For Special Surgery | 79 | 1.5 | 2556 | 0.12 |
| 6 | University Of Milan | 64 | 1.2 | 2418 | 0.07 |
| 7 | Rush University | 50 | 1.0 | 2271 | 0.06 |
| 8 | University Of Pittsburgh | 55 | 1.1 | 2049 | 0.04 |
| 9 | Nagoya University | 27 | 0.5 | 1927 | 0.01 |
| 10 | Seoul National University Snu | 46 | 0.9 | 1921 | 0.01 |
