# Supplementary material for: Platelet-rich plasma: A bibliometric and visual analysis from 2000 to 2022
Source: Medicine (Baltimore). 2024 Nov 15;103(46):e40530. doi: 10.1097/MD.0000000000040530 (PMC11575995; doi:10.1097/MD.0000000000040530)
Supplement: Supplementary file 4 [file medi-103-e40530-s004.docx]

Platelet-Rich Plasma：A Bibliometric and Visual Analysis from 2000 to 2022

Supplementary Tables

**Supplementary Table 4 Top 10 Co-cited authors in terms of the number of co-citations**

| Rank | Co-cited authors | Co-citations | | TLS | Institutions | Countries |
| --- | --- | --- | --- | --- | --- | --- |
| 1 | Anitua, Eduardo | | 2109 | 13994 | BTI Biotechnol Inst | Spain |
| 2 | Marx, Robert E. | | 1600 | 10808 | University of Miami | USA |
| 3 | Sanchez, Mikel | | 778 | 8563 | Hospital Vithas Vitoria | Spain |
| 4 | Filardo, Giuseppe | | 719 | 7976 | IRCCS Ist Ortoped Rizzoli | Italy |
| 5 | Kon, Elizaveta | | 618 | 6557 | Human Univ | Italy |
| 6 | Gentile, Pietro | | 592 | 2212 | Tor Vergata Univ | Italy |
| 7 | Weibrich, Gernot | | 564 | 5414 | Johannes Gutenberg Univ Mainz | Germany |
| 8 | Mishra, Allan | | 505 | 5486 | Stanford Univ | USA |
| 9 | Eppley, Barry L. | | 452 | 2956 | Eppley Plast Surg | USA |
| 10 | Ehrenfest, David M. | | 419 | 3961 | Dent Clin Throne | France |
