# Supplementary material for: Platelet-rich plasma: A bibliometric and visual analysis from 2000 to 2022
Source: Medicine (Baltimore). 2024 Nov 15;103(46):e40530. doi: 10.1097/MD.0000000000040530 (PMC11575995; doi:10.1097/MD.0000000000040530)
Supplement: Supplementary file 5 [file medi-103-e40530-s005.docx]

Platelet-Rich Plasma：A Bibliometric and Visual Analysis from 2000 to 2022

Supplementary Tables

**Supplementary Table 5 Top 10 authors with the highest number of publications**

| Rank | Authors | Records | Percentage (%) | H-index |
| --- | --- | --- | --- | --- |
| 1 | Anitua, Eduardo | 63 | 1.2 | 51 |
| 2 | Filardo, Giuseppe | 38 | 0.7 | 58 |
| 3 | Orive, Gorka | 33 | 0.6 | 59 |
| 4 | Kon, Elizaveta | 30 | 0.5 | 65 |
| 5 | Rodeo, Scott A. | 30 | 0.5 | 14 |
| 6 | Cole, Brian J. | 28 | 0.5 | 85 |
| 7 | Murray, Martha M. | 28 | 0.5 | 39 |
| 8 | Gentile, Pietro | 26 | 0.5 | 40 |
| 9 | Andia, Isabel | 24 | 0.4 | 34 |
| 10 | Marcacci, Maurilio | 23 | 0.4 | 67 |
