# Supplementary material for: Platelet-rich plasma: A bibliometric and visual analysis from 2000 to 2022
Source: Medicine (Baltimore). 2024 Nov 15;103(46):e40530. doi: 10.1097/MD.0000000000040530 (PMC11575995; doi:10.1097/MD.0000000000040530)
Supplement: Supplementary file 6 [file medi-103-e40530-s006.docx]

Platelet-Rich Plasma：A Bibliometric and Visual Analysis from 2000 to 2022

Supplementary Tables

**Supplementary Table 6 Top 10 most productive journals**

| Rank | Journals | Publications | IF (2021) | JCR Quartile | |
| --- | --- | --- | --- | --- | --- |
| 1 | American Journal of Sports Medicine | 166 | 7.01 | Q1 |  |
| 2 | Journal of Cosmetic Dermatology | 90 | 2.18 | Q3 |  |
| 3 | Knee Surgery Sports Traumatology Arthroscopy | 76 | 4.11 | Q1 |  |
| 4 | Platelets | 70 | 4.23 | Q3 |  |
| 5 | Journal of Orthopaedic Research | 63 | 3.10 | Q2 |  |
| 6 | Arthroscopy-The Journal of Arthroscopic and Related Surgery | 63 | 5.97 | Q1 |  |
| 7 | Journal of Endodontics | 59 | 4.42 | Q1 |  |
| 8 | Plos One | 54 | 3.75 | Q2 |  |
| 9 | International Journal of Molecular Sciences | 54 | 6.20 | Q1 |  |
| 10 | Journal of Oral and Maxillofacial Surgery | 48 | 2.13 | Q4 |  |
