# Supplementary material for: Platelet-rich plasma: A bibliometric and visual analysis from 2000 to 2022
Source: Medicine (Baltimore). 2024 Nov 15;103(46):e40530. doi: 10.1097/MD.0000000000040530 (PMC11575995; doi:10.1097/MD.0000000000040530)
Supplement: Supplementary file 7 [file medi-103-e40530-s007.docx]

Platelet-Rich Plasma：A Bibliometric and Visual Analysis from 2000 to 2022

Supplementary Tables

**Supplementary Table 7 Top ten most co-cited journals**

| Rank | Journals | Citations | IF(2021) | JCR Quartile | |
| --- | --- | --- | --- | --- | --- |
| 1 | American Journal of Sports Medicine | 9225 | 7.01 | | Q1 |
| 2 | Journal Of Bone and Joint Surgery-American Volume | 4253 | 6.55 | | Q1 |
| 3 | Arthroscopy-The Journal of Arthroscopic and Related Surgery | 3989 | 5.97 | | Q1 |
| 4 | Biomaterials | 3370 | 15.30 | | Q1 |
| 5 | Journal of Orthopaedic Research | 3302 | 3.10 | | Q2 |
| 6 | Journal of Periodontology | 3033 | 4.49 | | Q1 |
| 7 | Knee Surgery Sports Traumatology Arthroscopy | 2586 | 4.11 | | Q1 |
| 8 | Journal of Oral and Maxillofacial Surgery | 2485 | 2.13 | | Q4 |
| 9 | Clinical Orthopaedics and Related Research | 2453 | 4.83 | | Q1 |
| 10 | Plastic and Reconstructive Surgery | 2369 | 5.16 | | Q1 |
