# Supplementary material for: Platelet-rich plasma: A bibliometric and visual analysis from 2000 to 2022
Source: Medicine (Baltimore). 2024 Nov 15;103(46):e40530. doi: 10.1097/MD.0000000000040530 (PMC11575995; doi:10.1097/MD.0000000000040530)
Supplement: Supplementary file 8 [file medi-103-e40530-s008.docx]

Platelet-Rich Plasma：A Bibliometric and Visual Analysis from 2000 to 2022

Supplementary Tables

**Supplementary Table 8 Top 10 articles in terms of the number of Co-citations**

| Rank | | Articles | Author | Year | Co-citations |  |
| --- | --- | --- | --- | --- | --- | --- |
| 1 | | Platelet-rich plasma: growth factor enhancement for bone grafts | Marx Robert E | 1998 | 597 | |
| 2 | | Platelet-rich plasma: evidence to support its use | Marx Robert E | 2004 | 499 | |
| 3 | | Platelet-rich plasma (PRP): what is PRP and what is not PRP? | Marx Robert E | 2001 | 360 | |
| 4 | | Autologous platelets as a source of proteins for healing and tissue regeneration | Eduardo Anitua | 2004 | 332 | |
| 5 | Platelet-rich plasma: from basic science to clinical applications | | Foster Timothy E. | 2009 | 324 | |
| 6 | Platelet quantification and growth factor analysis from platelet-rich plasma: implications for wound healing | | Eppley Barry | 2004 | 302 | |
| 7 | Classification of platelet concentrates: from pure platelet-rich plasma (P-PRP) to leucocyte- and platelet-rich fibrin (L-PRF) | | Ehrenfest David M. | 2009 | 246 | |
| 8 | Plasma rich in growth factors: preliminary results of use in the preparation of future sites for implants | | Eduardo Anitua | 1999 | 222 | |
| 9 | Platelet-rich plasma injection for chronic Achilles tendinopathy: a randomized controlled trial | | De Vos Robert Jan | 2010 | 203 | |
| 10 | Treatment of Chronic Elbow Tendinosis with Buffered Platelet-Rich Plasma | | Mishra Allan | 2006 | 189 | |
|  |  | |  |  |  | |
