# Supplementary material for: Platelet-rich plasma: A bibliometric and visual analysis from 2000 to 2022
Source: Medicine (Baltimore). 2024 Nov 15;103(46):e40530. doi: 10.1097/MD.0000000000040530 (PMC11575995; doi:10.1097/MD.0000000000040530)
Supplement: Supplementary file 9 [file medi-103-e40530-s009.docx]

Platelet-Rich Plasma：A Bibliometric and Visual Analysis from 2000 to 2022

Supplementary Tables

**Supplementary Table 9 Top 10 most cited articles**

| Rank | Articles |  | Author | Year | Citations |
| --- | --- | --- | --- | --- | --- |
| 1 | Platelet-rich plasma: from basic science to clinical applications |  | Foster Timothy E. | 2009 | 741 |
| 2 | Platelet quantification and growth factor analysis from platelet-rich plasma: implications for wound healing |  | Eppley Barry | 2004 | 707 |
| 3 | A systematic review of the success of sinus floor elevation and survival of implants inserted in combination with sinus floor elevation |  | Pjetursson Bjarni E. | 2008 | 598 |
| 4 | Bone substitutes in orthopaedic surgery: from basic science to clinical practice |  | Campana Vincenzo | 2014 | 585 |
| 5 | Platelet-rich plasma injection for chronic Achilles tendinopathy: a randomized controlled trial |  | De Vos Robert Jan | 2010 | 527 |
| 6 | Treatment of chronic elbow tendinosis with buffered platelet-rich plasma |  | Mishra Allan | 2006 | 526 |
| 7 | Platelets, inflammation, and tissue regeneration |  | Nurden Alan T. | 2011 | 517 |
| 8 | Platelet lysates promote mesenchymal stem cell expansion: a safety substitute for animal serum in cell-based therapy applications |  | Doucet Christelle | 2005 | 498 |
| 9 | Efficacy and safety of corticosteroid injections and other injections for management of tendinopathy: a systematic review of randomized controlled trials |  | Coombes Brooke | 2010 | 483 |
| 10 | Therapy with platelet-rich plasma is more effective than placebo for knee osteoarthritis: a prospective, double-blind, randomized trial |  | Patel Sandeep | 2013 | 431 |
