# Supplementary material for: Platelet-rich plasma: A bibliometric and visual analysis from 2000 to 2022
Source: Medicine (Baltimore). 2024 Nov 15;103(46):e40530. doi: 10.1097/MD.0000000000040530 (PMC11575995; doi:10.1097/MD.0000000000040530)
Supplement: Supplementary file 10 [file medi-103-e40530-s011.docx]

Platelet-Rich Plasma：A Bibliometric and Visual Analysis from 2000 to 2022

Supplementary Tables

**Supplementary Table 10 Top 20 keywords with the highest frequency**

| Rank | Keywords | Occurrences | TLS |
| --- | --- | --- | --- |
| 1 | Platelet-Rich Plasma | 3871 | 13173 |
| 2 | Growth-Factors | 1428 | 6079 |
| 3 | Mesenchymal Stem-Cells | 1391 | 5943 |
| 4 | Regeneration | 441 | 2087 |
| 5 | Repair | 436 | 2122 |
| 6 | In-Vitro | 422 | 1922 |
| 7 | Injections | 394 | 1813 |
| 8 | Therapy | 384 | 1540 |
| 9 | Tendinopathy | 383 | 1840 |
| 10 | Osteoarthritis | 375 | 1992 |
| 11 | Proliferation | 363 | 1789 |
| 12 | Double-Blind | 323 | 1660 |
| 13 | Expression | 316 | 1241 |
